# Job preferences and trade-offs in rural health workforce retention: a discrete choice experiment from western China

**DOI:** 10.1093/heapol/czaf078

**Published:** 2025-10-18

**Authors:** Dongqiong Chen, Zigang Zhang, Sisi Ma, Jia Yin, Li Zhao, Lihua Jiang

**Affiliations:** Department of Health Policy and Management, West China School of Public Health and West China Fourth Hospital, Sichuan University, No. 17, Section 3, Renmin South Road, Wuhou District, Chengdu 610041, China; Department of Health Policy and Management, West China School of Public Health and West China Fourth Hospital, Sichuan University, No. 17, Section 3, Renmin South Road, Wuhou District, Chengdu 610041, China; Department of Health Policy and Management, West China School of Public Health and West China Fourth Hospital, Sichuan University, No. 17, Section 3, Renmin South Road, Wuhou District, Chengdu 610041, China; Human Resources Service Centre, Health Commission of Sichuan Province, No. 2 Yulin South Street, Wuhou District, Chengdu 610041, China; Department of Health Policy and Management, West China School of Public Health and West China Fourth Hospital, Sichuan University, No. 17, Section 3, Renmin South Road, Wuhou District, Chengdu 610041, China; Department of Health Policy and Management, West China School of Public Health and West China Fourth Hospital, Sichuan University, No. 17, Section 3, Renmin South Road, Wuhou District, Chengdu 610041, China; General Practice Ward/International Medical Centre Ward, General Practice Medical Centre, West China Hospital, Sichuan University, No. 37 Guoxue Alley, Wuhou District, Chengdu 610041, China; Teaching and Research Section of General Practice, the General Practice Medical Centre, West China Hospital of Sichuan University, No. 37 Guoxue Alley, Wuhou District, Chengdu 610041, China; Human Resources Department, West China Hospital, Sichuan University, No. 37 Guoxue Alley, Wuhou District, Chengdu 610041, China

**Keywords:** *Bianzhi*, primary healthcare workforce, discrete choice experiment, rural areas

## Abstract

The shortage and uneven distribution of primary healthcare workers in rural China have long persisted, with many studies focusing predominantly on salary and working conditions improvement. A discrete choice experiment involving 183 rural primary healthcare workers in Sichuan Province revealed the critical role of *Bianzhi* (a state-controlled employment system) in workforce retention. Findings demonstrated that *Bianzhi* dominated job preferences (*β*=0.964), with practitioners willing to sacrifice 18.2% of their monthly income to exchange for it. Beyond *Bianzhi*, near location, housing allowances, opportunities for continuing education, and children's education support significantly influenced job choices. Female workers exhibited 1.189 times greater sensitivity to workplace proximity than males (*P* < 0.001), while those with school-age children required 12.64% additional compensation for remote postings. Policy simulations indicated that combining *Bianzhi* with children's education support outperformed salary incentives alone. The study advocates optimizing rural healthcare workforce strategies by narrowing the gap between *Bianzhi* and *non-Bianzhi* positions, complemented by gender-sensitive and family-friendly measures. For other LMICs, it highlights the importance of understanding the true needs of health workers with different employment statuses.

Key messages

- *Bianzhi*, a state-guaranteed employment status, plays a dominant role in shaping primary healthcare workers’ job preferences. Policymakers could adapt certain benefits of the *Bianzhi* for *non-Bianzhi* positions to narrow the gap between them. Understanding what secure and insecure health workers truly need is the key to designing effective retention policies that could work in other LMICs
- Females and parents of school-age children tend to bear a responsibility of caregiving, influencing their job preferences. It is necessary to consider setting targeted subsidies such as childcare allowance, transportation allowance, and flexible work arrangements to help achieve social equity.
- Multidimensional policies can bring multiplicative effects than relying solely on salary. This can also provide LMICs with low-cost and efficient solutions for rural areas with limited resources.

## Introduction

Primary health workers (PHWs) are the backbone of a robust health system, serving as the first point of care for patients and ensuring the ‘last mile’ of healthcare delivery. However, China faces a significant challenge in the equitable distribution of its primary healthcare workforce, characterized by a stark disparity between the well-resourced eastern regions and the underserved western regions (Su et al. 2021). Western China, in particular, grapples with a persistent shortage of health professionals due to factors such as challenging geographical situations, economic underdevelopment, and a resulting lack of attractiveness for talent (Wang et al. 2012, Zhu et al. 2015). This enduring problem impedes progress towards universal health coverage and exacerbates health inequities.

To address this, China has implemented various policies. For example, the National Rural Doctor Program, launched in 2023, aims to recruit medical graduates for village clinics by offering incentives like student loan repayment (National Health Commission of the People's Republic of China 2023). At the provincial level, regions like Sichuan have pioneered models such as the County-level Integrated Health Alliance to promote resource sharing and personnel mobility (Health Commission of Sichuan Province 2024). Despite these top–down efforts, retaining skilled workers in rural areas remains a formidable challenge, compounded by an aging workforce and the continuous drain of highly-skilled health professionals to urban centres. Moreover, other policy innovations have been introduced, such as the ‘County-hired, township-used’ and ‘Township-hired, village-used’ modes. Under these schemes, a PHW may hold desirable employment status at a higher-level institution like a county-level hospital or a Community Health Centre (CHC) but be deployed to work long-term in a rural Township Health Centre (THC) or village clinic (National Health Commission of the People's Republic of China 2021). These polices, though implemented in a small group, vividly highlighted a universal trade-off faced by the entire rural health workforce: the need to weigh the employment security and tangible working conditions. This suggests a critical need not only to understand the issue from the perspective of rural PHWs—the very people these policies aim to attract and retain but also to formally quantify these trade-offs.

A critical factor in the career decisions of Chinese public-sector employees is the *Bianzhi* system. *Bianzhi* is a state-controlled employment quota system that often referred to as the ‘iron rice bowl’ (Liu et al. 2022), which provides not just a job, but a suite of lifetime benefits that are highly valued. For a rural PHW, this includes profound job security against dismissal, a stable salary and pension supported by government finance, superior social security coverage, and more clearly defined pathways for professional titles and career development (Shang et al. 2014, Tan 2021). This institutional arrangement has a profound impact on the recruitment, retention, and motivation of healthcare workers, making it a crucial variable to consider. While *Bianzhi* includes multiple benefits, it is typically perceived as a bundled, overall concept in real-world job decisions, and our study likewise treats it as such.

Discrete choice experiments (DCEs) are a powerful stated-preference method for quantifying such job preferences and have been widely applied to inform rural health workforce policies internationally. Global studies consistently demonstrate that while financial incentives are paramount, non-financial attributes such as manageable workloads, access to professional development, quality housing, and a supportive work environment are also crucial for retention (Henderson and Tulloch 2008, Mandeville et al. 2014, Mpembeni et al. 2015, Ajuebor et al. 2020, Lin et al. 2023). In China, a growing body of literature has adopted this methodology to explore health workforce preferences. Notably, the study (Bao and Huang 2021) confirmed that the immense value of *Bianzhi* in a DCE among medical students, highlighting its importance for the future workforce. Other studies have examined the preferences of different cadres, such as nurses (Song et al. 2015) or public health professionals (Cheng et al. 2024), or have explored preferences from the demand side, such as patients’ preferences for rural doctors (Fu et al. 2020, Liu et al. 2019).

However, despite the progress, a critical research gap remains. The existing literature in China has predominantly focused on the job preferences of medical students or recent graduates, who represent the future labor supply. While their intentions are important, they may not fully reflect the realities and priorities of experienced, in-service PHWs who are currently confronting the daily challenges of rural practice. The factors driving the retention of these seasoned professionals may differ significantly from those driving the initial recruitment of new graduates. To our knowledge, no prior study has specifically applied a DCE to quantify the job preference trade-offs among the existing PHWs in rural western China, a crucial group for ensuring the stability of the rural health system.

Therefore, this study aims to fill this gap by employing a DCE in this setting to quantify and understand the job preferences of currently serving primary healthcare workers. Specifically, our objectives are to measure the relative importance of *Bianzhi* compared to other key financial and non-financial incentives and analyse how job preferences vary across different demographic characteristics, providing evidence-based insights and targeted policies for developing more effective strategies to retain the existing rural health workforce.

## Methods

This study employed a DCE to quantitatively assess the job preference of rural PHWs in Sichuan, China. The DCE methodology is grounded in Random Utility Theory (McFadden 1972) and consumer preferences (Ryan et al. 2008, Holte et al. 2015, Bao et al. 2024), which posits that individuals make choices to maximize their utility. By asking participants to make repeated choices between hypothetical job scenarios, we can statistically estimate the relative importance they place on different job attributes. (Ryan et al. 2008).

### Study setting

This study was conducted in Sichuan Province, a region that serves as a representative microcosm of the healthcare workforce challenges faced by western China. As one of the country’s largest provinces, it is characterized by complex topography and significant socioeconomic disparities, which create immense difficulties in workforce management. These challenges can be reflected by the Health Personnel Density Index, ranging from a low of 0.18 in remote prefectures to 3.59 in the capital city of Chengdu (Zhou et al. 2022). Furthermore, the province confronts an urgent retention crisis, evidenced by an aging rural workforce where 35.3% of primary care physicians are over 55 years old in 2024, according to the unpublished data from the Sichuan Health Commission. This context makes Sichuan an ideal setting to investigate the preference of in-service rural PHWs and generate findings with high relevance for other regions facing similar issues.

### Attribute and level development

The identification of relevant attributes and their corresponding levels followed a rigorous, two-stage process recommended by the World Health Organization (WHO) (Ryan et al. 2012).

First, we conducted a systematic review of previous DCEs focused on healthcare workforce retention (Kolstad 2011, Rockers et al. 2012, Scott et al. 2013, Li et al. 2014, Song et al. 2015, Smitz et al. 2016, Bao and Huang 2021). An initial list of potential attributes was extracted from the literature. We made a decision to exclude broad attributes like work environment to focus on tangible, policy-amendable factors that governments and institutions can directly influence.

Second, the initial list was refined through expert consultations. To ensure policy relevance and practical grounding, the expert panel comprised three individuals with deep expertise in the field: two health policy researchers and one senior manager from a local health bureau with extensive experience in rural workforce management. The selection criteria for the panel were direct experience with health policy formulation or implementation in western China. The panel convened for a structured discussion to evaluate, consolidate, and finalize the seven key attributes most pertinent to rural PHWs in Sichuan.

Finally, to ensure the clarity and comprehensibility of the survey instrument, a pilot study was conducted with 10 health workers (not included in the final sample) from a nearby, non-sampled area. The pilot test confirmed that the attributes, levels, and choice tasks were clear and understandable. Feedback from the pilot led to minor wording refinements before the final survey was launched. The finalized attributes and their detailed description are shown in Table 1.

**Table 1.** Attributes and levels.

Attributes	Levels	Description
Salary	No change	Your monthly salary remains the same as your current salary.
	10% increase	A 10% increase on top of your current monthly salary.
	20% increase	A 20% increase on top of your current monthly salary.
Location	Remote	The facility is located >90 minutes travel time from the county town centre.
	Medium	The facility is located 30–90 minutes travel time from the county town centre.
	Near	The facility is located <30 minutes travel time from the county town centre.
Housing	None	No housing or subsidy is provided.
	Allowance provided	You receive a fixed monthly monetary subsidy for housing
	Housing provided	A furnished apartment/dormitory is provided by the facility, free of rent.
Facility quality	Basic	Equipped with all essential diagnostic tools and has a reliable drug supply.
	Good	Well-equipped with modern diagnostic tools and a comprehensive drug supply.
	Poor	Lacks some essential diagnostic equipment and has frequent drug stock-outs.
Opportunities for continuing education	None	No formal opportunities for off-site training or further education are provided.
	Less	Occasional (e.g. annual) opportunities for publicly funded off-site training.
	More	Regular, guaranteed opportunities for publicly funded off-site training and education.
Availability of *Bianzhi*	None	This is a contract-based position without formal government employment status.
	Provided	This position includes formal government employment status (*Bianzhi*) with associated security and benefits.
Support for children's education	None	No specific support is provided for your children's schooling.
	Provided	Your children are guaranteed priority enrolment in the county's upper-middle primary school.

### Experimental design and questionnaire

**Figure 1.**
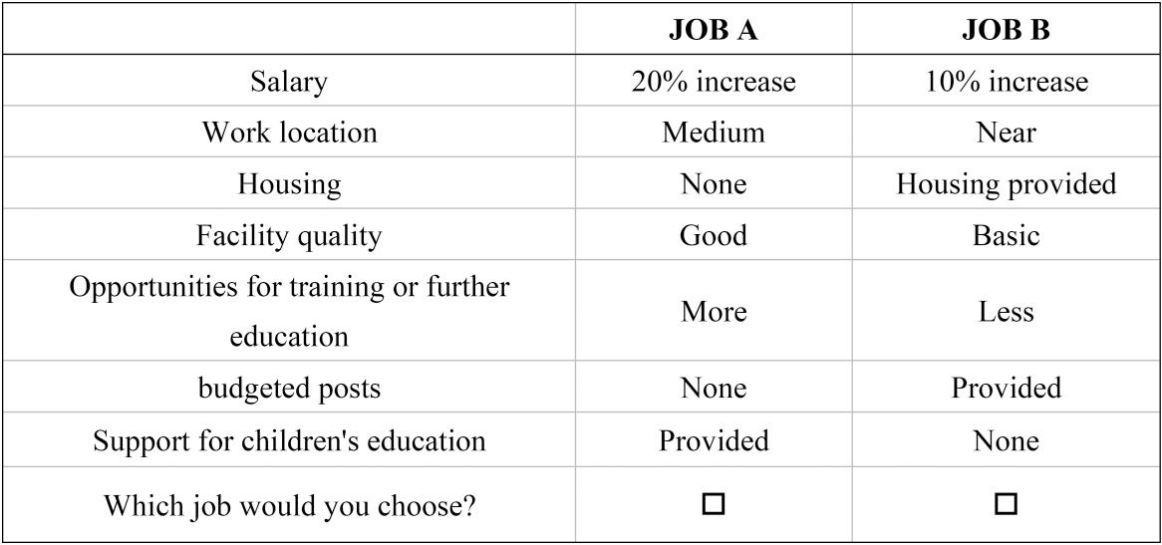
An example of a choice set.

The design was optimized using D-efficiency criteria to maximize the statistical efficiency of the experiment while maintaining a manageable cognitive burden on the respondents. The final design consisted of 12 choice sets using an orthogonal design to ensure balanced attribute-level combinations. Each choice set presented respondents with two hypothetical job positions (labelled Job A and Job B) and required them to select their preferred option. The 12 choice sets were determined to be appropriate to avoid respondent fatigue while collecting sufficient data for a robust analysis. This study did not include an opt-out option to obtain the maximum amount of information from each respondent. Figure 1 shows an example of a choice set.

### Survey administration and data collection

The survey was administered in person between August and September 2024. To ensure broad representation and logistical efficiency, data collection in each selected site was centralized. With the support of local health authorities, rural PHWs from various facility types, including the central township health centre itself, along with those from surrounding village clinics and community health centres, were invited to a designated township health centre to participate in the survey.

Each session began with a verbal explanation of the study’s background, objectives, and the confidential nature of their responses. The survey was administered in Mandarin. To ensure participants fully understood the task, the researcher first walked through a sample choice task, explaining how to compare the attributes of Job A and Job B and make a choice. *Bianzhi* is widely understood among healthcare professionals in China as a bundled package of employment benefits. No further decomposition was provided, as we aimed to assess the overall utility of this status. Participants were encouraged to ask questions before proceeding with the 12-choice tasks independently. The entire process for each participant took approximately 20–30 minutes.

### Sampling strategy and sample size

A multi-stage purposive sampling strategy was employed to ensure representation across Sichuan's diverse regions.

Firstly, the province was stratified into its five official economic zones: Chengdu Plain Economic Zone (central), Southern Sichuan Economic Zone, Northeastern Sichuan Economic Zone, Panxi Economic Zone, and Northwestern Sichuan Ecological Demonstration Zone. Secondly, one city/prefecture was purposively selected to represent its region’s typical economic and geographic characteristics from each zone, which are Chengdu (central), Yibin (southern), Nanchong (northern), Panzhihua (Panxi), and the Ganzi Prefecture (northwestern).

Finally, we targeted a mix of primary health facilities within each selected city/prefecture, including THCs, village clinics, and CHCs (administratively belonged to CHCs but are deployed to serve in grassroot facilities, as detailed policy above) to capture a comprehensive sample of rural PHWs. Full-time PHWs from these facilities who were available on-site were recruited via convenience sampling.

The required sample size was determined using the commonly cited rule of thumb by Orme and Johnson (Orme 1998, Johnson and Orme 2003):


$$N>\frac{500c}{t\times a}$$

Where *N* is the number of respondents, *t* is the number of choice tasks per respondent (12), *a* is the number of alternatives per task (2), and *c* is the maximum number of levels for any attribute (3× 3 = 9, considering interaction effects). Based on this formula, the minimum required sample size was 500 * 9/(12*2) = 187.5. Our target sample size of 200 participants (40 from each of the five sites) exceeds this minimum, ensuring sufficient statistical power for our analysis.

### Data analysis

#### Econometric modelling

Individuals are assumed to make choices based on utility maximization, and data from the DCE are analysed using this concept. The utility PHW *n* obtained from a specific job opportunity *m* consists of two components. The determined part $U_{ni}$ is a function of the observable job attribute ($x_{1}\cdots x_{m}$), each job attribute has its own corresponding weight ($\beta_{1}\cdots \beta_{m})$, and the random part $\varepsilon_{ni}$ is a function of unobservable job attributes and individual preference differences. As a result, utility from opportunity *i* can be expressed as:


(1) $$\begin{array}{rl} U_{ni}\;= & V_{ni}+\varepsilon_{ni}=\beta_{0}+\;\beta_{1}x_{1ni}+\beta_{2}x_{2ni}+\cdots +\beta_{m}x_{mni}\\ & +\varepsilon_{ni} \end{array}$$

Because utility cannot be measured directly, the coefficients of Equation (1) cannot be derived using direct estimation. However, DCE can address this problem. In DCEs, PHW *n* will be confronted with the given pairs of alternatives; hence, the probability of choosing alternative *i* over alternative *j* is given by:


(2) $$\begin{array}{rl} P_{ni} & =P_{r}(U_{ni}>U_{nj})=P_{r}(V_{ni}+\varepsilon_{ni}>V_{nj}+\varepsilon_{nj})\\ & =P_{r}(\varepsilon_{nj}-\varepsilon_{ni}>V_{nj}-V_{ni}) \end{array}$$

We first estimated a mixed logit model (MXL) to incorporate preference heterogeneity and then estimated a generalized multinomial logit (GMNL) to incorporate both preference and scale heterogeneity (Zeng 2024). Both models were estimated using 500 Halton draws. We chose the model with the best fit for the primary analysis. Specifically, we chose the superior model based on BIC rather than AIC because previous literature suggests that BIC is the most reliable criterion for selecting the correct model when modelling this type of heterogeneity in choice behaviour (Li et al. 2014). According to the BIC values, the MXL model is preferred over the GMNL model.

In the MXL model, the coefficient of the monetary attribute was fixed and continuous, the non-monetary coefficients were treated as random variables, and categorical variables were dummy coded. The value of the model's regression coefficient reflects the direction and magnitude of the influence of job attributes on job preference. A positive regression coefficient indicates that the factor has a positive effect on job preference, and vice versa. However, the magnitude is not an absolute value and has only relative significance.

#### Willingness to pay

Willingness to pay (WTP) estimates were calculated based on the regression results. As the salary variable is continuous and $x_{s}$ represents salary, the WTP for the other job attributes $x_{m}$ can be given by:


$$WTP(x)=-\frac{\partial U/\partial x_{m}}{\partial U/\partial x_{s}}=-\frac{\beta_{x_{m}}}{\beta_{x_{s}}}$$

where $\beta_{x_{m}}$ is the coefficient of the attribute *x_m_* from the regression model. WTP represents participants’ evaluation of the value of non-monetary job attributes, measured as the percentage of their average monthly salary. In this study, the positive coefficients should be interpreted as the willingness to forgo a certain amount of salary in exchange for another attribute, while the negative coefficient indicates the compensation that needs to be provided to the participant to accept the attribute.

#### Policy simulations

Policy simulations were conducted to demonstrate the predicted retention by changing job attribute levels. In this context, we utilized a logit model, the probability of choosing a job *i* is expressed as


$$P_{ni}=\frac{exp(V_{ni})}{\exp\;(V_{ni})+exp(V_{nj})}$$

Assuming that the job condition *i* is at the basic level, after improving a certain job attribute of the basic level *i*, the job condition beco. *j*, and this change in the probability of choosing the job *j*. becomes:


$$\Delta P=P_{ni}-P_{nj}$$

Differentiating the probability function with respect to variations in job attributes enables the forecasting of the effectiveness of diverse policies. This study uses the following as the baseline: keeping current income constant, no housing benefits or employee housing, basic facility quality, average continuing education opportunities, no *Bianzhi* provided, and no children’s education addressed.

Data were analysed using Stata version 17.

### Declaration of generative AI and AI-assisted technologies in the writing process

During the writing process of this work, the authors used ChatGPT to improve language and readability. After using this tool/service, the authors reviewed and edited the content as needed and take full responsibility for the content of the publication.

## Results

A total of 183 respondents were included in the study, with a valid response rate of 92.4% (183/198). Fifteen questionnaires were excluded due to incompleteness and illogicality issues. Data were collected from primary healthcare facilities in five sites, including 120 (65.6%) health workers from THC, 53 (29.0%) village doctors, and 10 (5.5%) workers from CHC. Table 2 presents the participants’ demographic characteristics. The average age of the participants was approximately 42 years, and 43.7% were female. They have an average of nearly 20 years of experience in the healthcare field and have spent an average of 13.6 years in their current rural areas.

**Table 2.** General demographic characteristics (*N* = 183).

Characteristics	*N* (%)
Gender	
Male	103 (56.3%)
Female	80 (43.7%)
Age	
<42 years old	81 (44.3%)
≥42years old	103 (56.3%)
Marital status	
Married	167 (91.3%)
Single, divorced, or widowed	16 (8.7%)
Educational level	
Associate bachelor degree and below	93 (50.8%)
Bachelor degree and above	90 (49.2%)
Professional title	
None or junior	95 (51.9%)
Medium or senior	88 (48.1%)
Job type	
Medical practitioners and assistant	105 (57.4%)
Registered nurses	24 (13.1%)
Rural doctors	34 (18.6%)
Technicians	5 (2.7%)
Pharmacist	5 (2.7%)
Others	10 (5.5%)
*Bianzhi* status	
Yes	126 (68.9%)
No	57 (31.2%)
Work time locally	
<14 years	97 (53.0%)
≥14years	86 (47.0%)
Separate from families	
Yes	69 (37.7%)
No	114 (62.3%)
Workers with school-aged child(ren)	
Yes	93 (50.8%)
No	90 (49.2%)

### Preferences for job attributes

Table 3 presents the regression results of the mixed logit model. Among all job attributes, *Bianzhi* commands the highest preference weight (*β* = 0.964, 95% CI = 0.743 to 1.185, *P* < 0.001). Near location (*β* = 0.451, 95% CI = 0.256 to 0.645, *P* < 0.001), housing allowance provision (*β* = 0.236, 95% CI = 0.054 to 0.418, *P* = 0.011), more opportunities for continuing education (*β* = 0.337, 95% CI = 0.163 to 0.511, *P* < 0.001), support for children’s education (*β* = 0.184, 95% CI = 0.051 to 0.317, *P* = 0.007) were preferred. Contrary to expectations, better facility quality was not a significant factor in preference (*β* = 0.126, 95% CI = −0.045 to 0.297, *P* = 0.148). This indicates that high-quality equipment and environment did not significantly influence the choice of these primary medical staff to accept a post in a rural area. Table 4 shows an analysis of the interaction effect on females. The effect on salary for females is not significantly different from that for males (*β* = 0.026, *P* = 0.108). Females prioritized being near the workplace significantly more than males (*β* = 1.189, *P* < 0.001), while remote posts significantly reduced their preferences (*β* = −0.668, *P* = 0.003). Providing housing subsidies (*β* = 0.431, *P* = 0.040) or direct housing (*β* = 0.504, *P* = 0.044) significantly increased the probability of women choosing. More opportunities for continuing education (*β* = 0.851, *P* = 0.001) and children's education welfare (*β* = 0.332, *P* = 0.048) significantly and positively influenced women's choices.

**Table 3.** Results of a mixed logit model.

	Coefficient	SD	95%CI	*P* value
Attribute				
Salary	0.053		(0.043, 0.063)	<0.001
Location (ref: medium)				
Remote	−0.367	0.611	(−0.552, −0.183)	<0.001
Near	0.451	0.535	(0.256, 0.645)	<0.001
Housing (ref: none)				
Allowance provided	0.236	−0.002	(0.054, 0.418)	0.011
Housing provided	0.214	−0.218	(0.026, 0.403)	0.026
Facility quality (ref: basic)				
Poor	−0.355	0.535	(−0.541, −0.170)	<0.001
Better	0.126	−0.348	(−0.045, 0.297)	0.148
Opportunities for continuing education (ref: fair)				
Fewer	−0.299	0.495	(−0.495, −0.103)	0.003
More	0.337	0.228	(0.163, 0.511)	<0.001
*Bianzhi* (ref: none)				
Provided	0.964	1.099	(0.743, 1.185)	<0.001
Support for children’s education (ref: none)				
Provided	0.184	0.358	(0.051, 0.317)	0.007
Model diagnostics				
Number of respondents	183			<0.001
Number of observations	4392			
Log likelihood	−1238.89			
Likelihood ratio χ^2^	113.53			

**Table 4.** Results of a mixed logit model (interaction with female).

	Coefficient	SD	95% CI	*P* value
Attribute				
Female*salary	0.026	0.075	(−0.006, −0.057)	0.108
Female*remote location (ref: medium)	−0.668	1.288	(−1.104, −0.232)	0.003
Female*near location	1.189	1.196	(0.623, 1.755)	<0.001
Female*housing allowance	0.431	0.015	(0.020, 0.843)	0.040
Female*housing provided	0.504	−1.331	(0.012, 0.996)	0.044
Female*poor facility quality	−0.619	1.522	(−1.130, −0.108)	0.018
Female*good facility quality	0.302	0.857	(−0.815, 0.090)	0.158
Female*fewer continuing education opportunities	−0.363	0.910	(−0.814, 0.090)	0.116
Female*more continuing education opportunities	0.851	0.837	(0.369, 1.333)	0.001
Female*Bianzhi provided	2.061	2.114	(1.315, 2.807)	<0.001
Female*children’s education support	0.332	−0.858	(0.003, 0.661)	0.048

*Interaction effect.

Table 5 shows an analysis of the interaction effect on *Bianzhi* status. This allows us to understand if the preferences of rural PHWs with *Bianzhi* differ significantly from those without. For rural PHWs currently without *Bianzhi* (the reference group), being offered a *Bianzhi* position yielded the highest utility (*β* = 1.644, *P* < 0.001). Several interaction terms were highly significant. Compared to their non-*Bianzhi* counterparts, PHWs who already have *Bianzhi* placed significantly higher value on salary increases (*β* = 0.046, *P* = 0.002), housing provision (*β* = 0.549, *P* = 0.001), and opportunities for career advancement (*β* = 0.685, *P* < 0.001). Furthermore, they showed a significantly stronger preference for working near home (*β* = 0.502, *P* = 0.001) and a greater aversion to working in remote locations (*β* = −0.557, *P* < 0.001).

**Table 5.** Results of a mixed logit model (interaction with a Bianzhi status).

	Coefficient	SD	95% CI	*P* value
Attribute				
Bianzhi*salary	0.046	0.092	(0.017, 0.074)	0.002
Bianzhi*remote location (ref: medium)	−0.557	0.914	(−0.858, −0.256)	<0.001
Bianzhi*near location	0.502	0.667	(0.207, 0.798)	0.001
Bianzhi*housing allowance	0.267	−0.019	(−0.018, 0.554)	0.067
Bianzhi*housing provided	0.549	−0.566	(0.237, 0.861)	0.001
Bianzhi*poor facility quality	−0.523	1.177	(−0.848, −0.200)	0.002
Bianzhi*good facility quality	0.101	0.792	(−0.183, 0.386)	0.486
Bianzhi*fewer continuing education opportunities	−0.379	0.774	(−0.687, −0.071)	0.016
Bianzhi*more continuing education opportunities	0.684	0.402	(0.367, 1.002)	<0.001
Bianzhi*Bianzhi provided	1.644	1.422	(1.222, 2.067)	<0.001
Bianzhi*children’s education support	0.301	0.729	(0.075, 0.526)	0.009

*Interaction effect.

### Willingness to pay

**Figure 2.**
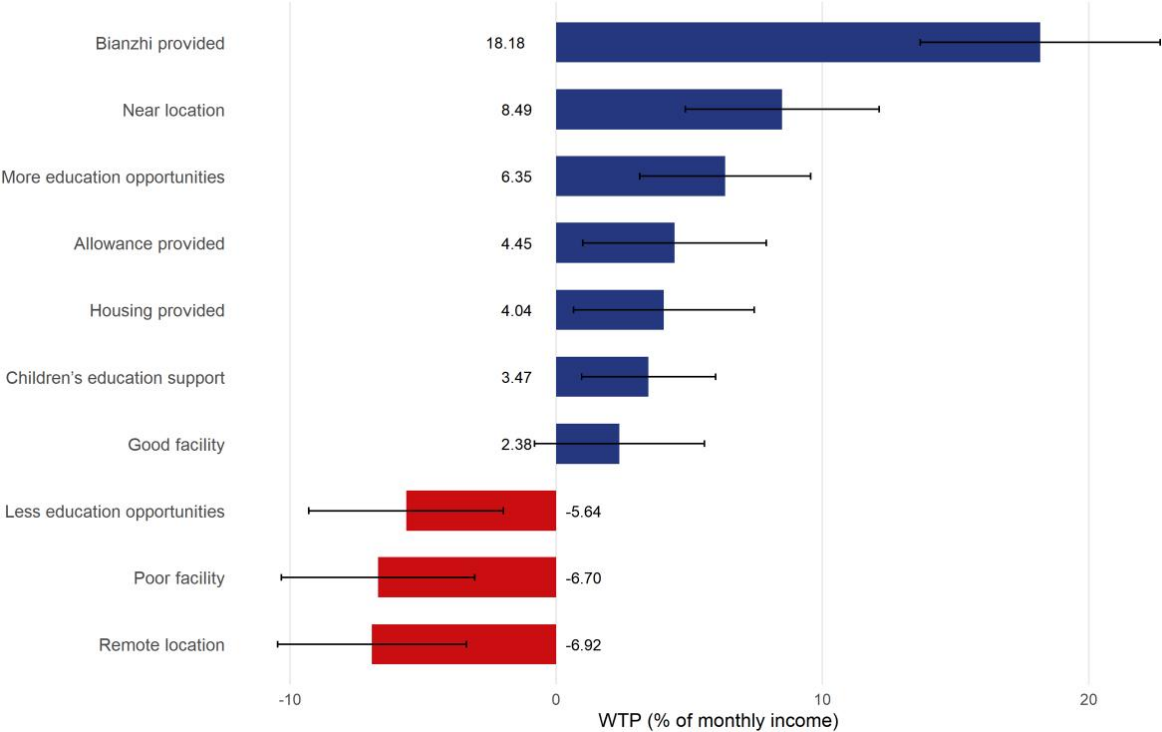
WTP estimates for the whole sample.

The WTP results are presented in Fig. 2. The results showed that *Bianzhi* commanded the highest premium, with workers willing to forgo 18.18% of their salary for it (*P* < 0.001), underscoring its institutional value in employment decisions. Location preferences were asymmetric, that is, workers required an 8.49% salary compensation for remote postings but accepted a 6.92% salary reduction for proximity (both *P* < 0.001). Similarly, more opportunities for continuing education were valued at a 6.35% salary trade-off, whereas limited opportunities required 5.64% compensation. The impact of facility quality was unidirectional, while poor facilities incurred significant disutility (requiring 6.70% compensation, *P* < 0.001), good facilities did not show a significant positive valuation.

**Figure 3.**
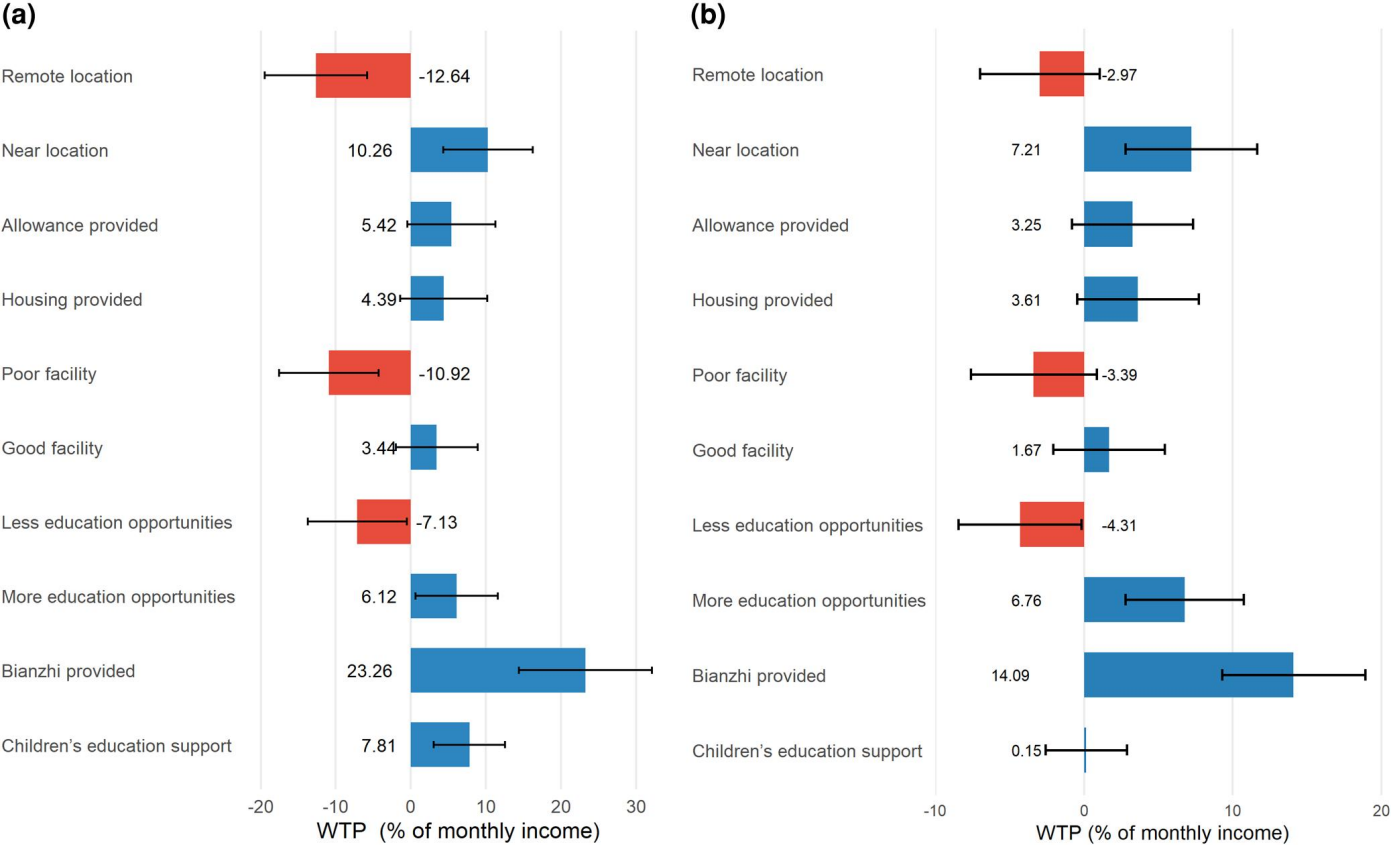
WTP estimates for workers with and without school-age children. (a) WTP for workers with school-age children. (b) WTP for workers without school-age children.

Figure 3 demonstrates the different WTP between workers with and without school-aged children. Workers with school-aged children exhibited acute location sensitivity were willing to require 12.64% compensation for remote posts and sacrifice 10.26% of their income for a nearby post. In contrast, workers without school-age children showed no significant aversion to remoteness (*P* = 0.147). While both groups prioritized *Bianzhi*, the valuation was higher among those with school-aged children. Families with school-aged children expressed a significant preference for educational support, which deserves to be paid 7.81% of their monthly salary. Both groups placed a similar value on more continuing education opportunities (6.12%–6.76%, *P* < 0.05). However, workers with school-aged children react more strongly to fewer continued education opportunities, requiring 65% higher compensation (−7.13% vs. −4.31%, *P* < 0.05). These results highlighted how childcare responsibilities amplify preferences for geographic accessibility, educational resources, and institutional benefits.

### Policy simulations

The analysis revealed that relying solely on salary increases yields diminishing marginal returns. A salary increase was predicted to boost job uptake probability by 52.5%. However, a subsequent 10% increase (to 20% total) resulted in a smaller additional gain of only 14.4%. Consequently, policies should not focus solely on monetary incentives. Instead, they need to be complemented by other non-monetary incentives to be more effective.

**Figure 4.**
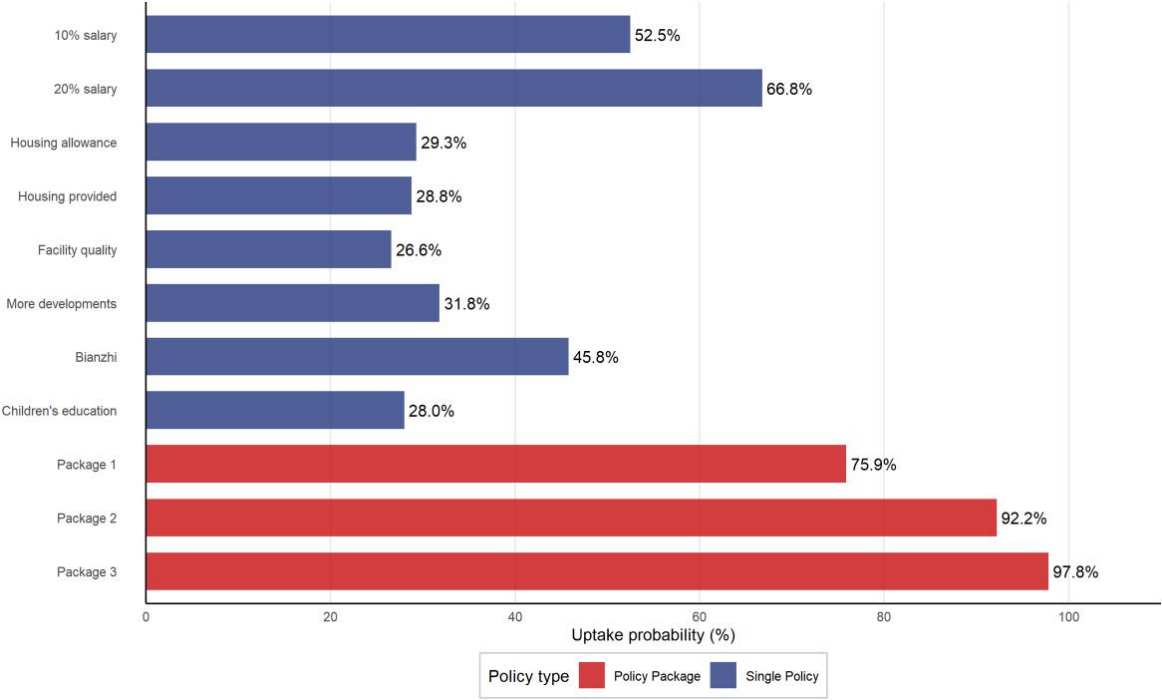
Policy simulation for various job postings (compared with a baseline job posting defined as: current salary; no allowance or housing provided; basic facility quality; keep opportunities for continuing education constant; no *Bianzhi* provided; no support for children’s education provided). Package 1: No salary increase + *Bianzhi* provided + Support for children’s education provided. Package 2: 10% increase of salary + *Bianzhi* provided + Support for children’s education provided. Package 3: 20% increase + *Bianzhi* provided + Support for children’s education provided.

In contrast, bundled policies eliminate diminishing returns. When *Bianzhi* was combined with education support (with no salary increase), the uptake probability increased to 75.9% (Package 1), significantly outperforming even a 20% salary increase (66.8%). By leveraging policy synergy, adding a 10% salary increase to this bundle (Package 2) further elevated the uptake probability to 92.2%. A 20% salary increase combined with the bundle achieved an uptake probability of 97.8% (Package 3; Figure 4).

## Discussion

### The dominant role of *bianzhi* in shaping PHW's job preferences

Retaining a skilled health workforce in rural areas is a persistent global challenge, with financial and non-financial incentives being widely studied across various international contexts. While our study focuses on the unique *Bianzhi* of China, it contributes important evidence about how a state-guaranteed employment mechanism shapes workforce preference and retention strategy.

Our primary finding confirms that *Bianzhi* is the most critical determinant in job selection for rural PHWs, a result consistent with prior studies on Chinese medical students (Bao and Huang 2021) and practicing physicians (Bao et al. 2023). Importantly, our interaction analysis shows that the role of *Bianzhi* differs between those who possess it and those who do not. This echoes the dual-track employment system in China, where workers and their motivations are stratified by their institutional identity (Qu et al. 2018). For non-*Bianzhi* rural PHWs, the system acts as a gateway to security, reflecting a strong desire to escape the precarity brought by contractual employment. Their preferences align with Maslow's hierarchy of needs (Maslow 1943), where safety and stability are fundamental concerns. This is further underscored by the insignificant preference for improved facility quality, despite evidence that patients strongly prefer well-equipped clinics (Liu et al. 2019). This contrast highlights that livelihood considerations outweigh workplace infrastructure in trade-offs. Conversely, for rural PHWs with *Bianzhi*, the system functions more as a platform for higher-order needs. Our interaction effect analysis confirms this pattern, showing that *Bianzhi* holders value career development and well-being more than their non-*Bianzhi* peers. While our analysis suggests PHWs with and without *Bianzhi* focus on different aspects of *Bianzhi*, for which potential explanations are offered, the relative importance of its individual components cannot be determined. Future research could build on our findings by decomposing these elements to provide more detailed policy guidance.

This leads to a vital policy implication. Given the fiscal constraints that make a large-scale expansion of the *Bianzhi* system unlikely, the policy focus should not merely be on allocating limited positions. Instead, a more sustainable strategy must be implemented to reduce the disparity between the two tracks systematically. Concrete measures include providing stable, long-term contracts, enforcing ‘equal pay for equal work’, guaranteeing equal access to continuing education, and investing in tangible benefits like housing support. While the *Bianzhi* system is not replicable outside of China, the secure employment arrangements can fundamentally alter employee preferences. The core lesson is understanding what health workers under different employment statuses truly need, which is the key to designing effective retention policies that could be adapted in other LMICs.

### Gender- and family-responsive interventions are critical to address subgroup disparities

This study revealed gender disparities and parental childcare in location preferences among rural health workers. Female workers showed 1.189 times higher sensitivity to workplace proximity than their male counterparts. Previous research has consistently shown that women often bear a disproportionate share of caregiving responsibilities, such as childcare and eldercare, which can significantly influence their job preferences (Boniol et al. 2019, UN Women 2019). The need to balance caregiving duties likely makes female workers more inclined to choose work arrangements that allow them to be closer to home and more readily available for caregiving (Deole et al. 2023).

Furthermore, workers with school-age children showed amplified sensitivity to location, requiring a 12.64% compensation to accept remoteness, compared to a 10.26% willingness to sacrifice income for proximity. In contrast, those without children displayed no significant aversion to location. This can also be interpreted by the childcare responsibility restriction. The lack of short-term and rental housing for remote postings further exacerbates recruitment challenges (Bryan et al. 2024).

Addressing these issues requires policymakers to consider gender- and family-responsive interventions, such as implementing targeted financial incentives for remote postings, with differentiated allowances, including subsidized childcare, housing, and transportation allowances, coupled with flexible work arrangements. Studies have proposed that policies addressing gender-specific needs can enhance employee well-being (Wang et al. 2025). This approach also aligns with the WHO’s recommendation to employ a tailored package in rural and remote areas (World Health Organization 2021).

### Multidimensional incentives have multiplicative effects

Combining *Bianzhi* with education support (Package 1: 75.9%) outperformed a 20% salary increase alone (66.8%); the synergy between *Bianzhi*, children's education support, and salary in policy simulations raised uptake probability to approximately 90%, demonstrating how non-monetary factors can reduce reliance on financially unsustainable salary hikes.

However, the practical feasibility of implementing such a comprehensive package warrants careful consideration. Expanding *Bianzhi* is constrained by strict government quotas and significant long-term fiscal commitments. Similarly, providing direct ‘children's education support’ can be administratively complex, requiring cross-departmental coordination between health and education bureaus. Therefore, while our findings point to an ideal combination, policymakers must navigate these under real-world constraints. For example, where creating new *Bianzhi* posts is not achievable, key components of *Bianzhi,* such as long-term contracts and/or pension (or other benefits) could be offered separately as a partial substitute to enhance the attractiveness of non-*Bianzhi* positions. Education support could be implemented as a direct financial subsidy for school fees or transportation, which is less complex than guaranteeing school placement. Furthermore, these high-value, resource-intensive packages could be strategically targeted at the most remote locations, rather than being universally applied, thereby maximizing their cost-effectiveness.

This approach of bundling holistic packages that address both professional stability and family welfare resonates with global evidence. Studies in other LMICs have shown that integrated support systems can improve health worker retention more effectively than isolated financial incentives (Russell et al. 2021, Jinah et al. 2024). For instance, in Australia, providing guaranteed paid locum relief for 6 weeks every 12 months was more effective in retaining General Practitioners (GPs) than a 50% increase in retention payments or 20% rural skills loading (Russell et al. 2021). This implication also resonates with the WHO’s recommendation to implement policies being bundled in rural and remote areas (World Health Organization 2021).

### Strengths and limitations

The main strength of this study is that the DCE introduces new perspectives on various job attributes, unlike previous research that examined factors in isolation. We combined traditional and institutional incentives, filling the cognitive gap in the existing literature on the role of institutional factors (*Bianzhi*) in rural human resource management in developing countries. The existing literature fails to recognize gender- and family-based sensitivity to workplace proximity. We extend the depth of preference heterogeneity and propose targeted strategies to provide a basis for accurate policy design and achieving social equity. It also provides evidence of low-cost and efficient solutions for rural areas with limited resources; however, the cost-benefit analysis should be quantified in the future.

However, our study has several important limitations. First, the process of attribute development could have been more inclusive. The attributes were selected based on a literature review and a pilot test of the questionnaire. We excluded important domains such as work environment and organizational culture to focus on direct and actionable policies. Moreover, rural PHWs were not formally involved in co-designing the attributes and levels. This lack of direct respondent input in the design phase may limit the comprehensive realism of the job scenarios presented.

Second, there may be a potential risk of confirmation bias associated with the inclusion of *a priori Bianzhi* attribute, while this pre-determination was based on real-world and literature-based evidence. In addition, the sample size of *Bianzhi’*s interaction analysis was uneven between groups.

Finally, there was a slight discrepancy between the final and target sample sizes due to the logistical challenges of reaching health workers in the remote and sparsely populated Ganzi Prefecture. The study captures preferences at a single time point, neglecting the longitudinal change in priorities because rural PHWs’ preferences can change under policy changes and societal developments. Therefore, future research should attempt to track how the effectiveness of incentives changes over time.

## Conclusions

This study examined primary healthcare workers’ preferences for various job attributes in rural areas. Our results confirm the dominant role of Bianzhi, while also revealing that workers with and without Bianzhi emphasize different components of this bundled employment status. Gender- and family-sensitive policies also emerged as important for tailoring retention strategies. These insights provide actionable evidence for policymakers in China and may offer useful evidence for other health systems.

## Data Availability

The datasets used and/or analysed during the current study are available from the corresponding author on reasonable request.

## Reflexivity statement

The authors include five women and one man, we have made efforts to ensure that the research recognize and address the different needs and challenges faced by various gender group. Our authors’ group consists of both senior researchers in health policy and staff dedicated to human resources, which bring fresh insights and innovative ideas. Regional location is another important dimension that should be considered. Sichuan Province is a multi-ethnic, multi-topographic region with huge differences in economic development, subjects is also from different zones and ethnics, and this diversity is much representative.

## Abbreviations

PHWs: Primary Health Workers
WHO: the World Health Organization
DCE: Discrete Choice Experiment
MXL: Mixed Logit Model
GMNL: Generalized Multinomial Logit
WTP: Willingness to pay
THC: Township Health Centre
CHC: Community Healthcare Centre
LMICs: Low- and middle-income countries
GPs: General Practitioners

## References

[czaf078-B1] Ajuebor O, Boniol M, McIsaac M et al Increasing access to health workers in rural and remote areas: what do stakeholders’ value and find feasible and acceptable? Hum Resour Health 2020;18:77. 10.1186/s12960-020-00519-2 33066792 PMC7565226

[czaf078-B2] Bao ML, Huang CR. Job preferences of medical and nursing students seeking employment in rural China: a discrete choice experiment. BMC Med Educ 2021;21:146. 10.1186/s12909-021-02573-3 33673842 PMC7934374

[czaf078-B3] Bao ML, Huang CR, Wang L et al Eliciting primary healthcare physicians’ preferences for job characteristics in rural China: a discrete choice experiment. BMJ Open 2023;13:e056741. 10.1136/bmjopen-2021-056741 PMC10030470 36921936

[czaf078-B4] Bao ML, Huang CR, Wang HX. Advances in the application of discrete choice experiments in the field of human resources for health [in Chinese]. Chinese General Practice 2024;27:3184–91. 10.12114/j.issn.1007-9572.2022.0664

[czaf078-B5] Boniol M, McIsaac M, Xu L et al Gender Equity in the Health Workforce: An Analysis of 104 Countries. Geneva: World Health Organization, 2019. https://www.who.int/publications/i/item/gender-equity-in-the-health-workforce-analysis-of-104-countries (25 July 2025, date last accessed).

[czaf078-B6] Bryan W, Kasey DS, Tianna F. Rural workforce recruitment and retention factors (Policy Brief). 2024. https://www.ruralhealth.us/nationalruralhealth/media/documents/advocacy/nrha-policy-brief-workforce-retention-factors-final-3-7-25.pdf (30 October 2025, date last accessed).

[czaf078-B7] Cheng HZ, Tian RT, Chen DQ et al Job preferences of master public health candidates in Northeast China based on discrete choice experiments. BMC Health Serv Res 2024;24:1291. 10.1186/s12913-024-11810-6 39468610 PMC11520076

[czaf078-B8] Deole SS, Deter M, Huang Y. Home sweet home: working from home and employee performance during the COVID-19 pandemic in the UK. Labour Econ 2023;80:102295. 10.1016/j.labeco.2022.102295 36440260 PMC9678226

[czaf078-B9] Fu PP, Wang Y, Liu SM et al Analysing the preferences for family doctor contract services in rural China: a study using a discrete choice experiment. BMC Fam Pract 2020;21:148. 10.1186/s12875-020-01223-9 32711467 PMC7382837

[czaf078-B10] Health Commission of Sichuan Province . Notice on Issuing the Implementation Plan for Comprehensively Promoting the Construction of Close-Knit County-level Medical and Health Communities in Sichuan Province. 2024. https://wsjkw.sc.gov.cn/scwsjkw/qtwj/2024/9/25/b52609cbb858411bb7e6cff227fa9c54.shtml (25 July 2025, date last accessed).

[czaf078-B11] Henderson LN, Tulloch J. Incentives for retaining and motivating health workers in Pacific and Asian countries. Hum Resour Health 2008;6:18. 10.1186/1478-4491-6-18 18793436 PMC2569066

[czaf078-B12] Holte JH, Kjaer T, Abelsen B et al The impact of pecuniary and non-pecuniary incentives for attracting young doctors to rural general practice. Soc Sci Med 2015;128:1–9. 10.1016/j.socscimed.2014.12.022 25569609

[czaf078-B13] Jinah N, Adnan IK, Bakit P et al Retention strategies for medical doctors in low- and middle-income countries (LMICs): are they effective? A scoping review. BMC Health Serv Res 2024;24:1662. 10.1186/s12913-024-12154-x 39734187 PMC11684273

[czaf078-B14] Johnson R, Orme B. Getting the Most from CBC. Sawtooth software research paper series (Research Paper Series). 2003. https://sawtoothsoftware.com/resources/technical-papers/getting-the-most-from-cbc (25 July 2025, date last accessed).

[czaf078-B15] Kolstad JR . How to make rural jobs more attractive to health workers. Findings from a discrete choice experiment in Tanzania. Health Econ 2011;20:196–211. 10.1002/hec.1581 20094993

[czaf078-B16] Li JH, Scott A, McGrail M et al Retaining rural doctors: doctors’ preferences for rural medical workforce incentives. Soc SciMed 2014;121:56–64. 10.1016/j.socscimed.2014.09.053 25306410

[czaf078-B17] Lin TK, Werner K, Kak M et al Health-care worker retention in post-conflict settings: a systematic literature review. Health Policy Plan 2023;38:109–21. 10.1093/heapol/czac090 36315458 PMC9849712

[czaf078-B18] Liu SM, Gu YY, Yang Y et al Tackling brain drain at Chinese CDCs: understanding job preferences of public health doctoral students using a discrete choice experiment survey. Hum Resour Health 2022;20:46. 10.1186/s12960-022-00743-y 35606873 PMC9125964

[czaf078-B19] Liu Y, Kong QX, de Bekker-Grob EW. Public preferences for health care facilities in rural China: a discrete choice experiment. Soc Sci Med 2019;237:112396. 10.1016/j.socscimed.2019.112396 31404884

[czaf078-B20] Mandeville KL, Lagarde M, Hanson K. The use of discrete choice experiments to inform health workforce policy: a systematic review. BMC Health Serv Res 2014;14:367. 10.1186/1472-6963-14-367 25179422 PMC4161911

[czaf078-B21] Maslow AH . A theory of human motivation. Psychol Rev 1943;50:370–96. 10.1037/h0054346

[czaf078-B22] McFadden D. (Ed.) Conditional Logit Analysis of Qualitative Choice Behavior. Berkeley, CA: Institute of Urban and Regional Development, 1972.

[czaf078-B23] Mpembeni RNM, Bhatnagar A, LeFevre A et al Motivation and satisfaction among community health workers in Morogoro Region, Tanzania: nuanced needs and varied ambitions. Hum Resour Health 2015;13:44. 10.1186/s12960-015-0035-1 26044146 PMC4458000

[czaf078-B24] National Health Commission of the People's Republic of China . Guiding Opinions on Carrying out the Work of “County Hiring Township Employment” and “Township Hiring Village Employment” for Health Talents. 2021. https://www.nhc.gov.cn/jws/c100073/202112/db67a3729aa74fea959f2da124cc1bb1.shtml (25 July 2025, date last accessed).

[czaf078-B25] National Health Commission of the People's Republic of China . Notice on the Implementation of the Special Program for College Students to Become Rural Doctors. 2023. https://www.nhc.gov.cn/jws/c100073/202304/26fb5311dee74b45bef6b9bb375626c1.shtml (25 July 2025, date last accessed).

[czaf078-B26] Orme B . Sample size issues for conjoint analysis studies (Technical Paper Series). 1998. https://content.sawtoothsoftware.com/assets/dd3f6a38-285f-441f-a88c-678d7c8aaffb (25 July 2025, date last accessed).

[czaf078-B27] Qu J, Fu C, Wen X. Changes in labor relations in the dual-track system reform. In: Organizational Transition and Systematic Governance: Labor Relations in Enterprises. Singapore: Springer Singapore, 2018, 79–122.

[czaf078-B28] Rockers PC, Jaskiewicz W, Wurts L et al Preferences for working in rural clinics among trainee health professionals in Uganda: a discrete choice experiment. BMC Health Serv Res 2012;12:212. 10.1186/1472-6963-12-212 22824497 PMC3444383

[czaf078-B29] Russell D, Mathew S, Fitts M et al Interventions for health workforce retention in rural and remote areas: a systematic review. Hum Resour Health 2021;19:103. 10.1186/s12960-021-00643-7 34446042 PMC8393462

[czaf078-B30] Ryan M, Gerard K, Amaya MA. *Using Discrete Choice Experiments to Value Health and Health Care*, *Vol. 11*. Dordrecht, The Netherlands: Springer, 2008, 13–46. 10.1007/978-1-4020-5753-3_1

[czaf078-B31] Ryan M, Kolstad JR, Rockers PC et al How to Conduct a Discrete Choice Experiment for Health Workforce Recruitment and Retention in Remote and Rural Areas: a User Guide with Case Studies. Washington, DC: World Bank, 2012. http://documents.worldbank.org/curated/en/586321468156869931 (25 July 2025, date last accessed).

[czaf078-B32] Scott A, Witt J, Humphreys J et al Getting doctors into the bush: general practitioners’ preferences for rural location. Soc Sci Med 2013;96:33–44. 10.1016/j.socscimed.2013.07.002 24034949

[czaf078-B33] Shang J, You L, Ma C et al Nurse employment contracts in Chinese hospitals: impact of inequitable benefit structures on nurse and patient satisfaction. Hum Resour Health 2014;12:1. 10.1186/1478-4491-12-1 24418223 PMC3896777

[czaf078-B34] Smitz MF, Witter S, Lemiere C et al Understanding health workers’ job preferences to improve rural retention in Timor-Leste: findings from a discrete choice experiment. PLoS One 2016;11:e0165940. 10.1371/journal.pone.0165940 27846242 PMC5112867

[czaf078-B35] Song KM, Scott A, Sivey P et al Improving Chinese primary care providers’ recruitment and retention: a discrete choice experiment. Health Policy Plan 2015;30:68–77. 10.1093/heapol/czt098 24357198

[czaf078-B36] Su BB, Liu SJ, Lu YJ et al Evaluation of human resource allocation of primary healthcare in China: based on agglomeration degree [in Chinese]. Chinese Journal of Health Policy 2021;14:49–54. 10.3969/j.issn.1674-2982.2021.04.007

[czaf078-B37] Tan QC . The nature, behavior and development of primary health care institutions [in Chinese]. Acad China 2021;8:195–209. 10.3969/j.issn.1002-1698.2021.08.020

[czaf078-B38] UN Women . Progress of the World's Women 2019–2020: Families in a Changing World. New York: UN Women, 2019. https://www.unwomen.org/sites/default/files/Headquarters/Attachments/Sections/Library/Publications/2019/Progress-of-the-worlds-women-2019-2020-en.pdf (25 July 2025, date last accessed).

[czaf078-B39] Wang ML, Narcisse MR, Rodriguez K et al Gender disparities in job flexibility, job security, psychological distress, work absenteeism, and work presenteeism among US adults. SSM Popul Health 2025;29:101761. 10.1016/j.ssmph.2025.101761 40007632 PMC11850157

[czaf078-B40] Wang J, Zhao Y, Hao YL et al The present situation,problems and recommendations of rural health care personnel construction in China [in Chinese]. Chin J Health Policy 2012;5:45–51. 10.3969/j.issn.1674-2982

[czaf078-B41] World Health Organization . WHO guideline on Health Workforce Development, Attraction, Recruitment and Retention in Rural and Remote Areas [Internet]. Geneva: World Health Organization, 2021. https://www.ncbi.nlm.nih.gov/books/NBK570762/. (25 July 2025, date last accessed). 34057827

[czaf078-B42] Zeng T . Frequentist model averaging in the generalized multinomial logit model. Comput Stat 2024;39:605–27. 10.1007/s00180-022-01306-4

[czaf078-B43] Zhou MH, Tan H, He SZ. Analysis on the implementation and equity of health human resource allocation plan in Sichuan Province during the ‘13th five-year plan’ [in Chinese]. Soft Science of Health 2022;36:55–60. 10.3969/j.issn.1003-2800.2022.10.011

[czaf078-B44] Zhu XL, Chen QK, Yang SX. The current situation and problems of primary health care personnel since the new round of China's health care reform [in Chinese]. Chin J Health Policy 2015;8:57–62. 10.3969/j.issn.1674-2982.2015.11.012

